# The detection of influenza virus at the community pharmacy to improve the management of local residents with influenza or influenza-like disease

**DOI:** 10.1186/s40780-017-0091-x

**Published:** 2017-08-08

**Authors:** Akio Kawachi, Yusuke Sakamoto, Shunya Mouri, Mitsuaki Fukumori, Riku Kawano, Takaya Murakami, Junichiro Sonoda, Keiko Narumi, Yoshihiro Shimodozono, Kenji Etoh, Susumu Chiyotanda, Takashi Furuie, Keizo Sato, Masao Fukumori, Toshiro Motoya

**Affiliations:** 1 Graduate School of Pharmaceutical Sciences, Kyushu University of Health and Welfare, Miyazaki, Japan; 2 Tomitaka Pharmacy, Miyazaki, Japan; 3 Havas Worldwide Japan K.K, Tokyo, Japan; 4 Hyuga-city and Higashi-usuki County Pharmaceutical Association, Miyazaki, Japan; 5 Chiyoda Hospital, Miyazaki, Japan; 6 Hyuga-city Healthcare Center, Miyazaki, Japan

**Keywords:** Point-of-care testing, Influenza, Pharmacist, Community pharmacy

## Abstract

**Background:**

As of 2014, community pharmacies in Japan are approved by the Ministry of Health, Labour and Welfare to measure lipid panel, HbA1c, glucose, ALT, AST and γ-GTP, but not to screen for influenza virus. We provided influenza virus screening tests at a community pharmacy to triage people with symptoms suggestive of influenza. Participants were given appropriate advice on how to prevent the spread of and safeguard against influenza. We subsequently evaluated the effects of community pharmacy-based influenza virus screening and prevention measures.

**Methods:**

Local residents with symptoms suggestive of influenza participated in this study. Influenza virus screening tests using nasal samples were provided to the pharmacy, and we assessed samples for the presence of influenza virus. The study consisted of a preliminary interview, informed consent, and screening test on Day 1, and mail-in survey on Day 14.

**Results:**

A total 52 local residents participated in the study. The number of participants and influenza virus positive results followed the same trend as the influenza epidemic in the study area. Influenza virus was found in 28.8% of samples. There was no significant difference between the appearance ratios of subjective symptoms among influenza-positive and influenza-negative groups. The percentages of participants who were first screened at the pharmacy, and those who were first screened at a clinic and then tested again at the pharmacy, were 71.2% (37/52) and 28.8% (15/52), respectively. In the latter group, 14 of 15 were negative by screening at the clinic, and one was diagnosed with influenza without testing. Subsequently, 46.8% (7/15) of participants tested positive for influenza by pharmacy-based screening. According to the mail-in survey, all influenza-positive (100%, 7/7) and 35.3% (6/17) of influenza-negative participants visited the clinic after being tested at the community pharmacy; test results between the community pharmacy and clinic were consistent. A total 64.7% (11/17) of symptomatic participants who tested negative recovered spontaneously at home.

**Conclusions:**

Implementation of influenza virus screening followed by provision of appropriate advice for both influenza-positive and influenza-negative participants at the community pharmacy showed a significant effect on improving the health of the local community.

## Background

Point-of-care testing (POCT) is generally defined as medical testing performed near the time and place of patient care [1]. POCT is considered to be important for improving medication adherence and enhancing patient understanding [2]. A revision of the 2014 act on the clinical laboratory technicians authorizes measurement of lipid panel, HbA1c, glucose, ALT, AST and γ-GTP at community pharmacies in Japan [3]. Whereas POCT affords community pharmacists the opportunity to detect lifestyle diseases such as diabetes, dyslipidemia, and others, screening for infectious diseases that affect millions of Japanese each year [4], such as influenza, are not approved at community pharmacies in Japan.

Influenza is a contagious and acute respiratory disease caused by the influenza virus. Morbidity, mortality, and the economic and social costs of influenza cannot be managed without controlling the extent and severity of annual influenza epidemics [5]. In general, influenza is more severe than the common cold but the two have similar symptoms; therefore, it can be difficult to distinguish them based on subjective symptoms. It is considered that efficient detection of influenza virus infection by use of rapid test kits, followed by recommendations for clinical consultation and prevention, will improve community health with respect to influenza.

We provided influenza virus screening tests at a community pharmacy, to triage people with symptoms suggestive of influenza, and provided appropriate advice on how to prevent the spread of and safeguard against influenza. We subsequently evaluated the effects of community pharmacy-based influenza virus screening.

## Methods

Participants included local residents of Hyuga City in Miyazaki Prefecture, Japan, who had symptoms suggestive of influenza and volunteered to participate in the study. Influenza virus screening tests were provided to Tomitaka Pharmacy from 1 December 2015 to 31 March 2016, to cover the influenza epidemic season in Japan. Participant samples were collected and testing performed in the “measurement office” at the community pharmacy. We used the Fuji DRICHEM IMMUNO AG1 analyzer (Fujifilm, Tokyo, Japan) and IMMUNO AG immunochromatographic assay kit to detect the presence of influenza virus in participant specimens.

**Fig. 1:**
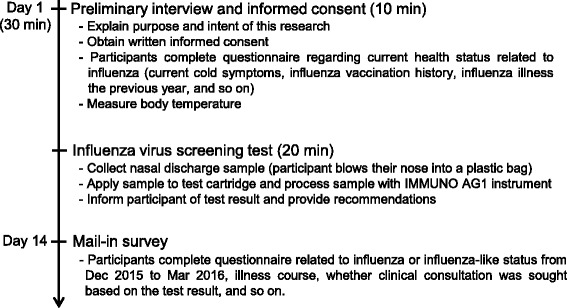
Schematic of community pharmacy-based influenza virus screening

A schematic of the pharmacy-based screening is shown in Fig. 1. The screening process consisted of a preliminary interview, obtaining informed consent, and testing on Day 1, followed by a mail-in survey on Day 14. Prior to sample collection, the researchers and pharmacists explained the purpose and intent of the study to potential participants. After obtaining their written informed consent, a nasal discharge sample was collected from each participant and stored in a plastic bag. The specimen was then mixed with sample buffer and loaded onto an IMMUNO AG cartridge FluAB, according to the manufacturer’s instructions [6]. While the test was being processed, participants were given questionnaires to complete querying their current health status, vaccination status and so on.

**Fig. 2:**
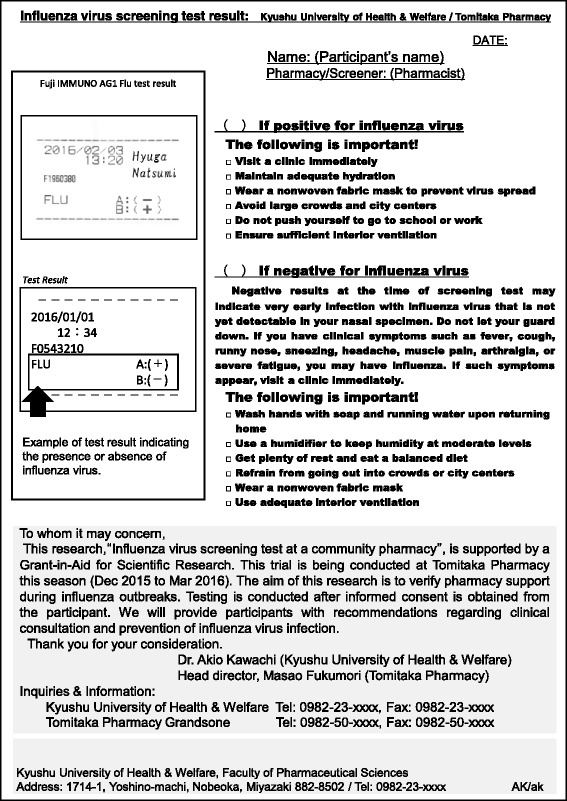
Sample reporting chart of influenza virus screening test result

An example of a screening test report is shown in Fig. 2. This chart contains the test result and pharmaceutical or medical advice for the participant, according to “Comprehensive countermeasures against influenza this winter” of the Ministry of Health, Labour and Welfare [7].

Follow-up self-completed questionnaires were distributed to participants querying their course of illness as of Day 14. The response rate for the mail-in survey was 46.2% (24/52). Statistical analyses were performed using IBM SPSS Statistics Version 21.0 (IBM Corp., Armonk, NY, USA). *P*-values less than 0.05 were considered statistically significant using Fisher’s exact test.

## Results

A total of 52 local residents of Hyuga participated in this study. Participant characteristics, health history, and current health status are shown in Table 1. The proportions of men and women were 40.4% and 59.6%, respectively. The median age was 30.5 years, and participants aged 10–49 years accounted for approximately 80% of the total. No underlying disease was reported by 71.2% of participants. The percentage of participants with symptoms suggestive of influenza was 96.2%, and 46.2% and 53.8% reported being vaccinated and unvaccinated, respectively.

**Table 1:** Participant characteristics, health history, and current health status

Variables		
Sex, n (%)		
Male	21	(40.4%)
Female	31	(59.6%)
Age, y		
Median, range (min, max)	30.5	(0, 81)
Age structure, n (%)		
0–9 years	2	(3.8%)
10–19 years	17	(32.7%)
20–29 years	6	(11.5%)
30–39 years	5	(9.6%)
40–49 years	13	(25.0%)
50–59 years	7	(13.5%)
Over 60 years	2	(3.8%)
Underlying diseases, n (%)		
None	37	(71.2%)
Diseases reported (multiple answers allowed)	7	(13.5%)
Bronchial asthma (6), hypertension (4), allergic rhinitis (1), emphysema (1), lumbar disc hernia (1), rheumatoid arthritis (1)		
Unknown	8	(15.4%)
Subjective symptoms ^a^ of influenza or common cold, n (%)		
No symptoms	2	(3.8%)
Positive for symptoms	50	(96.2%)
Flu vaccination status		
Vaccinated	24	(46.2%)
Unvaccinated	28	(53.8%)

^a^Subjective symptoms of influenza or common cold include fever, headache, arthralgia, myalgia, severe fatigue, cough, runny nose, sneezing, and others

**Fig. 3:**
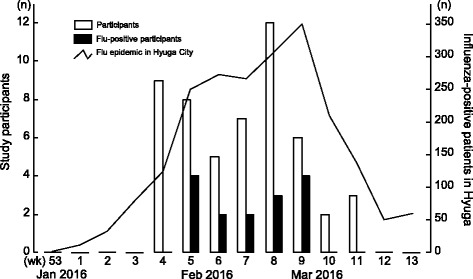
Results of influenza virus screening at community pharmacy. The number of participants (*open bars*) and number of influenza-positive participants (*solid bars*) were shown along with the epidemic status of healthcare center precincts in Hyuga City. All positive participants had type B influenza

The results of influenza virus screening at the community pharmacy are shown in Fig. 3. An influenza epidemic in Hyuga healthcare center precincts began in week 1 of 2016 and peaked at week 9. In line with the influenza epidemic, the number of study participants increased from week 4 through week 11. Participants who tested positive for influenza virus were observed from weeks 5 to 9 of 2016. Influenza virus was found in 28.8% of participants, and all were subtype B.

**Fig. 4:**
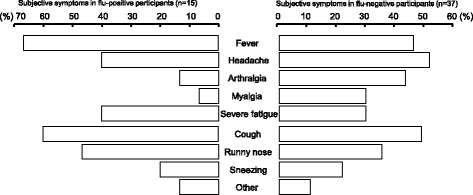
Appearance ratios of subjective symptoms in influenza-positive and negative groups

The appearance ratios of subjective symptoms among influenza-positive and negative groups are shown in Fig. 4. Typical influenza and cold symptoms were observed among participants in both groups. The percentages of fever, severe fatigue, cough, and runny nose in the influenza-positive group were higher than those of the negative group, but the difference was not significant. The percentages of headache, arthralgia, myalgia, and sneezing among participants in the influenza-positive group were not higher than those in the negative group.

**Fig. 5:**
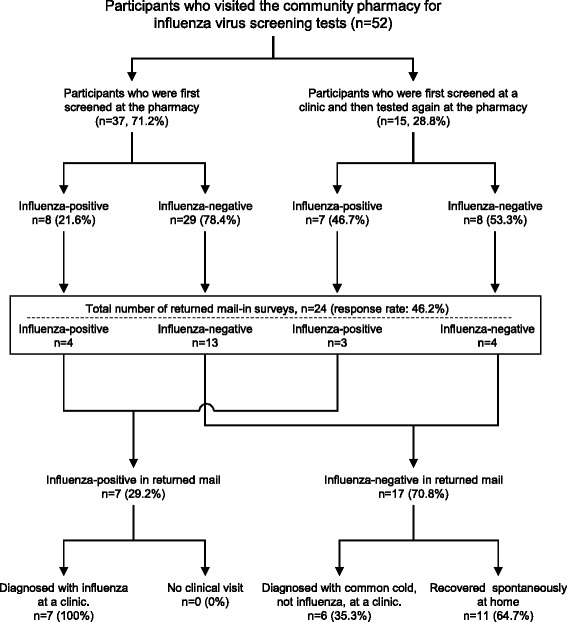
Profile of the participant disposition

As shown in Fig. 5, the percentages of participants who were first screened at the pharmacy, and those who were first screened at a clinic and then tested again at the pharmacy, were 71.2% (37/52) and 28.8% (15/52), respectively. In the former group, 21.6% (8/37) of participants had positive screening test results and 78.4% (29/37) had negative results. In the latter group, 14 of 15 participants were negative upon screening at the clinic, with one participant diagnosed with influenza with no testing. Upon subsequent screening at the community pharmacy, 46.7% (7/15) of participants were positive for influenza virus and the other 53.3% (8/15) were negative. In addition, 24 of all 52 participants responded to the mail-in survey. All (100%, 7/7) participants who were positive for influenza and 35.3% (6/17) who were negative had a clinical consultation after being tested at the community pharmacy; test results between the community pharmacy and clinic were consistent. In addition, 64.7% (11/17) of symptomatic participants who tested negative for influenza reported that they recovered spontaneously at home. Overall, 91.7% (22/24) of participants indicated their satisfaction with the community pharmacy-based influenza virus screening.

## Discussion

POCT has been approved to be performed by community pharmacists since 2014 in Japan. Advanced pharmacies measure lipid panel, HbA1c, glucose, ALT, AST, and γ-GTP, but do not perform influenza virus screening. In this study, we conducted influenza virus screening at a community pharmacy in Japan.

The weekly number of participants in screening followed the trend of an influenza epidemic in the study area. During the study period, there were 52 participants, with 28.8% (15/52) testing positive for influenza. Participants who tested positive were recommended to visit a clinic immediately. All influenza-positive participants who responded to a subsequent mail-in survey had had a clinical consultation after screening at the pharmacy. Influenza virus testing at the clinic confirmed the results of testing at the pharmacy. This shows the definite effects of community pharmacy-based influenza virus screening for improving community health, with pharmacists leading the early detection and treatment of influenza. All participants in the pharmacy screening, whether positive or negative for influenza, were provided with additional recommendations on their test result report. It is especially important for people who test negative for influenza to understand that a negative result on an influenza virus detection test may indicate that it is too early in the course of infection to detect influenza virus in nasal samples. Indeed, nearly half of participants who were tested at the pharmacy after obtaining a negative test result at a clinic were positive for influenza by pharmacy screening. Therefore, careful and continuous follow-up is necessary to detect influenza infection, even if an individual has been diagnosed with common cold and not influenza. The influenza A virus (AH1pdm09 subtype) outbreak of 2009 [8] resulted in disruption of public health services, such as overloading of emergency departments [9]. Screening and monitoring of influenza by pharmacists in the community during the influenza epidemic season may be helpful to prevent such disruption to health services and clinics.

Influenza and the common cold have similar symptoms, which include fever, headaches, muscle or body aches, severe fatigue, cough, and runny or stuffy nose. However, the consequences of influenza are more serious. We found no significant difference between the appearance ratios of subjective symptoms among participants in the influenza-positive and negative groups. It is clearly difficult to distinguish these two types of illness. However, recommendations given by community pharmacists to local residents about preventive measures and when to visit their local clinic can have additional positive benefits for community health.

According to the 2015–2016 influenza virus surveillance in Japan [10], influenza A virus (AH1pdm09) demonstrated an upward trend, and the detection ratio of influenza B virus exceeded that of AH1pdm09 from week 3 in 2016. In this study, we observed the presence of influenza B virus alone among participants, but not influenza A virus, from weeks 5 to 9 of 2016. It has been speculated that based on the duration of influenza B detection, the epidemic influenza type and area where the virus is prevalent coincides with a higher detection ratio of influenza B virus.

In the present study, the amount of participants aged over 60 years were quite small compared to other age groups. Presumably, one of the reasons may be associated with age-specific influenza morbidity that the patients of aged over 60 years was only 11% of estimated cumulative patients with influenza during the 2015–2016 epidemic season [10]. And also the circumstances that most elderly people with underlying diseases are managed mainly by hospitals, and influenza vaccination campaign for the elderly at high risk of developing severe complications is conducted by the Ministry of Health, Labor and Welfare might be considered as other reasons.

Overall, the community pharmacy may play an increasingly important role in the management of patients with influenza or influenza-like disease. The feasibility of influenza virus screening test at community pharmacies in Japan is expected to be enhanced.

## Conclusion

Implementation of influenza virus screening tests at community pharmacies, followed by appropriate recommendations for both influenza-positive and -negative individuals, could have a significant effect on the improvement of community health. This activity is expected to become a new role for community pharmacists.

## Abbreviations

ALT: alanine aminotransferase
AST: aspartate aminotransferase
HbA1c: hemoglobin A1c
γ-GTP: gamma-glutamyl transpeptidase
POCT: point of care testing
